# Uncontrolled chronic rhinosinusitis with and without polyps is predicted by T cell subtype

**DOI:** 10.1186/2045-7022-5-S4-O9

**Published:** 2015-06-26

**Authors:** Tanja Soklic Kosak, Mira Silar, Izidor Kern, Irena Hocevar Boltezar, Peter Korosec

**Affiliations:** 1 Department of Otorhinolaryngology and Head & Neck Surgery, University Medical Centre Ljubljana, Ljubljana, Slovenia; 2 University Clinic of Respiratory and Allergic Diseases Golnik, Laboratory for Clinical Immunology and Molecular G, Golnik, Slovenia; 3 University Clinic of Respiratory and Allergic Diseases Golnik, Cytology and Pathology Laboratory, Golnik, Slovenia; 4 Department of Otorhinolaryngology and Head & Neck Surgery, University Medical Centre Ljubljana, Department of Otorhinolaryngology and Head & Neck, Ljubljana, Slovenia

## Background

Patients with uncontrolled CRS have persistent bothersome symptoms despite appropriate treatment with nasal corticosteroids and endoscopic sinus surgery. The aim of this study was to identify which T cell subtype of untreated, steroid naive CRSwNP and CRSsNP patients influences the disease control.

## Methods

CRS was diagnosed based on symptoms, nasal endoscopy and CT scan. Sinonasal mucosa (uncinate process) or middle meatal polyp tissue were obtained from 31 untreated CRS patients (18 CRSwNP and 13 CRSsNP) and analyzed histopathologicaly and by flow cytometry. Patients were subsequently treated medically and if unresponsive surgically. The average follow up duration was 15,8 ± 4,6 months after tissue sampling. Patients with persistent symptoms on VAS ≥5 despite treatment represented the uncontrolled CRS group.

## Results

The CRSwNP group had significantly more CD4+ cells then CRSsNP group (34,05±19.89 vs. 22,04±16.71, p=0.028) and significantly less CD8+ cells then CRSsNP group (56,94±11.76 vs. 67.15±23.22, p=0.002). All uncontrolled CRSwNP patients had tissue eosinophilia. In the well-controlled CRSwNP there were significantly more Th17 CD4+CCR6+ cells (13.28±5.96) then in the uncontrolled CRSwNP (7.16±4.89), p=0.046. In the uncontrolled CRSwNP there were significantly more double negative T17 CD4-CD8-CCR6+ cells (12.20; IQR 2.20-21.8) then in the well-controlled CRSwNP group (5.40; IQR 1.65-9.15), p=0,010. In the uncontrolled CRSsNP group there were significantly more cytotoxic CD3+CD8+ cells (79.86±7.86) then in the well-controlled CRSsNP group (52.95±28.17), p= 0.045. In the well-controlled CRSsNP group there were significantly more double negative CD4-CD8- cells (46.93±28.29) then in the uncontrolled CRSsNP group (20.70±7.59), p = 0.019.

## Conclusions

By flow cytometry we were able to show the Th17 CD4+CCR6+ cell to predict well-controlled CRSwNP; we could explain that by Th17 cell plasticity, being able to transform into Th1 cell. Double negative T17 CD4-CD8-CCR6+ cell (also capable of IL-17 production) predicted uncontrolled CRS; double negative T17 cells are known to promote immune cell recruitment and tissue immunoglobulin production in autoimmune diseases. In CRSsNP being cytotoxic type of inlammation the Tc CD8+ cell predicted the uncontrolled disease. The double negative CD4-CD8- predicted the well-controlled CRSsNP which could be the result of CD8 silencing.

